# The Frequency of Mutations in Exon 11 of the c-kit Gene in Patients With Leukemia

**DOI:** 10.5505/tjh.2012.60320

**Published:** 2012-03-15

**Authors:** Syed Rizwan Hussain, Sunil G Babu, Hena Naqvi, Pradyumn Singh, Farzana Mahdi

**Affiliations:** 1 Era’s Lucknow Medical College and Hospital, Lucknow, India; 2 Babasaheb Bhimrao Ambedkar University, Lucknow, India

**Keywords:** C-kit, Lösemi, SSCP, Mutasyon

## Abstract

**Objective:** To determine the frequency of mutations in exon 11 of the c-kit gene in patients with leukemia.

**Material and Methods:** The study included 50 leukemia patients (31 with acute myeloid leukemia, 5 with acutelymphoblastic leukemia, 9 with chronic myeloid leukemia, and 5 with chronic lymphocytic leukemia) that underwentPCR-SSCP, followed by direct DNA sequencing.

**Results:** In all, 28 of the leukemia patients were male and 22 were female, with a mean age of 31.88 years (range: 2-65years). In total, 20 mutations in 19 patients were identified, including Lys550Asn, Tyr568Ser, Ile571Thr, Thr574Pro,Gln575His, Tyr578Pro, Asp579His, His580Gln, Arg586Thr, Asn587Asp, and Arg588Met, as well as novel point mutationsat codons Ile563Lys, Val569Leu, Tyr570Ser, and Pro577Ser. Ile571Leu substitution was observed in 2 patients andTrp582Ser substitution was observed in 3 patients.

**Conclusion:** The results suggest that mutations in exon 11 of the c-kit gene might be useful as molecular geneticmarkers for leukemia

## Abstract (tr)

**Amaç:** Lösemili hastalarda c-kit geni ekson 11 mutasyonlarının sıklığını belirlemek.

Gereç ve Yöntemler: Bu çalışma PCR-SCCP ve sonrasında direkt DNA sekanslama yapılan 50 lösemi hastası (31 akut myeloid lösemi, 5 akut lenfoblastik lösemi, 9 kronik myeloid lösemi ve 5 kronik lenfositik lösemi) hastası ile yapıldı.

**Bulgular:** Genel olarak lösemi hastalarının 28’i erkek ve 22’si kadındı ve ortalama yaş 31,88 (aralık: 2-65) yıldı. Toplam olarak 19 hastada Lys550Asn, Tyr568Ser, Ile571Thr, Thr574Pro, Gln575His, Tyr578Pro, Asp579His, His580Gln, Arg586Thr, Asn587Asp ve Arg588Met ve ayrıca Ile563Lys, Val569Leu, Tyr570Ser ve Pro577Ser kodonlarında yeni nokta mutasyonları olmak üzere 20 mutasyon saptandı. Ile571Leu yerine konması 2 hastada ve Trp582Ser yerine konması 3 hastada görüldü.

**Sonuç:** Sonuçlar c-kit geni ekson 11 mutasyonlarının lösemi için moleküler genetik işaretler olarak faydalı olabileceğini düşündürmektedir.

## INTRODUCTION

Leukemia is a heterogeneous disease characterized byhematopoietic progenitor cells that acquire genetic lesions,leading to loss of differentiation, an increase in selfrenewal,and unregulated proliferation. In 2000, approximately256,000 children and adults worldwide developedsome form of leukemia, and 209,000 died due to leukemia,which accounts for about 3% of the almost 7 millioncancer-related deaths and about 0.35% of all deathsthat year. A study that examined the incidence of canceraccording to 16 sites of the body reported that leukemiawas the 12thmost common class of neoplastic disease andthe 11th most common cause of cancer-related death [1].

Leukemia is classified based on the presence of specificcytogenetic abnormalities, as well as the French-American-British (FAB) classification of leukemic cells [2].A number of studies suggest that c-kit—a member of thetype III receptor tyrosine kinase (RTK) family is importantfor the development of a range of cells including hematopoieticcells, and plays a role in leukemogenesis—[3].High-level expression of c-kit has been reported in 60%-80% of acute myeloid leukemia (AML ) patients [4,5] andpoint mutation of c-kit has been observed in 33.35%45%of AML patients [6]. Nevertheless, many of these studieswere screened for c-kit mutations, only in a limited portionof the c-kit coding sequence and others were limitedby small study populations.

It is known that c-kit is a leukemia proto-oncogeneand that activated c-kit mutations are likely to contributeto the development of leukemia in humans [7,10].The activation sphere of the c-kit receptor has resulted inconstitutive c-kit kinase activity and c-kit receptors thatharbor such mutations when introduced into mammaliancells in the downstream signaling pathways lead to factorindependentgrowth in vitro and leukemogenesis in vivo[7,11]. The c-kit gene is a member of the type III TKR family,which includes platelet-derived growth factor receptors(PDGFRs) [12,13,14].

Type III TKRs share a sequence homology and a similarstructure, with 5 immunoglobulin-like repeats in theextracellular domain, 1 transmembrane domain (TM),1 juxtamembrane domain (JM), 2 intracellular tyrosinekinase domains (TK1 and TK2) divided by a kinase insertdomain (KI), and 1 C-terminal domain [15]. The genomiclocus that encodes the c-kit gene receptor has 21 exons,ranging from 100 bp to 300 bp [16]. Mutations in exon11 of the c-kit gene have been reported in gastrointestinalstromal tumors, solid tumors, and germ cell tumors [17-19]. To date, no study has reported the frequency or prevalenceof mutations in exon 11 of the c-kit gene in leukemiapatients in India. As such, the present study aimed toidentify mutations in exon 11 of the c- kit gene in Indianpatients with malignant leukemias (acute myeloid leukemia[AML ], acute lymphoblastic leukemia [ALL], chronicmyeloid leukemia [CML ] and chronic lymphocytic leukemia[CLL ]) and to determine if c-kit gene mutations couldbe used as molecular genetic markers for leukemia.

## MATERIALS AND METHODS

**Patients**

The study included 50 leukemia patients and 50healthy controls. Ethical approval of the study protocolwas granted by the Era’s Lucknow Medical College andHospital Ethics Committee. Clinical data of patients aswell as control samples were recorded. Blood or bone marrowsamples were stained according to the Leishman stainmethod and the patients were classified according to FABcriteria [20] as follows: AML (n = 31); ALL (n = 5); CML(n = 9); CLL (n = 5).

**DNA Extraction**

Specimens were collected from 50 routinely processedunstained bone marrow slides and blood diagnosed asleukemic by the hospital’s hematology department andwere then stored at –20 ° C. Genomic DNA was extractedaccording to Moskaluk et al. 1997 [21] with some modification.Lysis buffer and proteinase K (10 mg mL–1) wereadded to samples, followed by incubation at 55 °C for 1-2h. Then, 10 μL of 10% SDS, 120 μL of 5M NaCl, and 300μL of RN Ase-free water were added, followed by thoroughmixing and shaking. Next, 400 μL of phenol:chloroform(4:1) was added, followed by centrifugation at 15,000 rpmfor 10 min at 4 °C. The supernatant was collected into anew tube. For precipitation chilled absolute alcohol wasadded and centrifuged at 11,000 rpm for 5 min at 4 °C.The precipitate was washed with 70% alcohol and dissolvedin 100 μL of HPLC water.

**Polymerase Chain Reaction and Single-StrandConformational Polymorphism**

Polymerase chain reaction (PCR) was performedwith 25 μL of PCR reaction mixture containing 200 ngof template DNA, 10 pmol of each primer, 10 mmol L–1of each mix dNTP, 1X reaction buffer, and 0.3 U of Taqpolymerase enzyme (Fermentas, Germany) in an MJ Minithermocycler (Bio-Rad, UK). The cycling conditions wereas follows: 35 cycles of denaturation at 94 °C for 30 s,followed by annealing at 56 °C for 30 s, and extensionat 72°C for 30 s, followed by a final extension step at72 °C for 10 min using the following primers [19]: forward:5-ATTATTAAAAGG TGATCTATTTTTC-3; reverse:5-ACTGTTATGTGTACCCAAAAAG-3. Single-strand conformationalpolymorphism analysis was performed accordingto Orita et al. [22] with some modifications. Sampleswere denatured at 94° C for 8 min, and then immediatelytransferred to ice. Then 15 μL of amplified PCR productwas loaded along with 15 μL of denaturing dye on 8%polyacrylamide gel. The gel was run in pre-cooled 1XxTBE buffer. The gel tank was placed in a cold room (4°C) and run for 15 h at 140 V. The PCR product on thegel was silver stained after electrophoresis. Electrophoresismobility shifts in the patients’ single-stranded or doublestranded PCR products were detected via comparison withthe controls’ PCR products that were run in adjacent lanes.

**Sequencing Analysis**

Amplicons were sequenced using an automatedsequencer (ABI 3730XL DNA Analyzer, AppliedBiosystems, Foster City, California, USA) and examinedusing FinchTV software. Amplicons with mutations werereconfirmed via sequencing in both directions and in independentsecond samples. Sequences were analyzed usingBioEdit JustBio software.

## RESULTS

**Figure 1:**
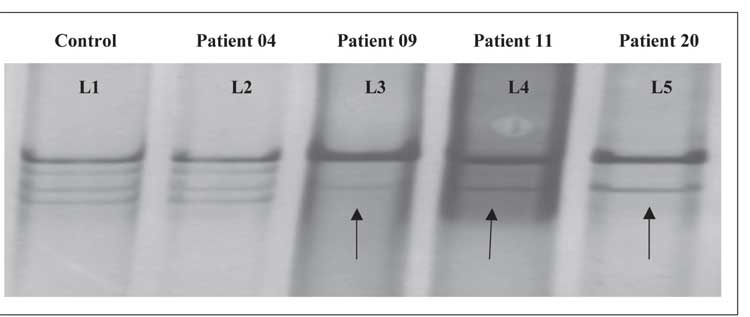
SSCP-PAGE analysis of exon 11 (257 bp) shows electrophoresis mobility shift on native page. Lane 1 is the control, andlanes 2-5 are patients (no shift in patient 04, and a shift in patients 09, 11, and 20).

Among the 50 leukemia patients 28 were male and 22were female, with a mean age of 31.88 years (range: 2-65years). The patients were classified according to FAB criteria[20] as AML (n = 31), ALL (n = 05), CML (n = 09),and CLL (n = 05). A shift in position was noted in 39 ofthe 50 patient samples via native SSCP-PAGE. In 19 of the31 AML cases 20 point mutations were observed, whereasnone were detected in the ALL , CML , or CLL patients.Point mutation details are shown in Figure 1 and Table 1.

## DISCUSSION

The present study is the first to report mutations inexon 11 of the c-kit gene in leukemia patients fromNorthern India. Previous molecular studies have reportedseveral mutations in exon 11 in different types of tumors.Mutations in exons 9, 13, and 17 of the c-kit gene areless frequently detected than those in exon 11. Thesemutations are considered rare in gastrointestinal stromaltumors, with a reported frequency of <10%, but are notuncommon in hematopoietic malignancies and germ cellneoplasms [23-25]. It was reported that 65%-92% of gastrointestinalstromal tumors harbor kit-activating mutations,the majority of which are localized at the juxtamembraneregion involving exon 11 [26,27].

**Figure 2:**
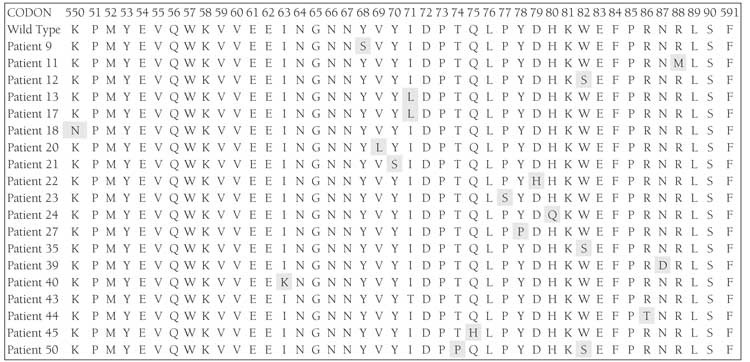
Amino acid sequences of exon 11 of the c-kit gene. The sequence starts at codon 550 and ends at 591. The wild-typesequence is shown above. Point mutations are shown in

The majority of exon 11 mutations are clustered withinthe classical hotspot region of the 50 end involving codons550-560; however, a second hotspot at the 30 end involvingcodons 576-590 has been described by Antonescuet al. [28], which includes frame deletions of 1 to severalcodons (typically involving codons 557-560) andpoint mutations and internal tandem duplications (typicallyinvolving the 30 end). In the present study heterogeneouspoint mutations in AML patients were observed,some of which were and were not previously reported.In 19 of the 31 AML patients 20 point mutations wereobserved; a point mutation at Lys550Asn in 1 patientand at Ile571Leu in 2 other patients were previouslyreported [29-32]. Mutation at codon 582 (Trp582Tyrand Trp582His) was reported by Tae Won Kim et al. [30]and Ying-Yong Hou et al. [17] reported Trp582Try andTrp582Gln; however, in 3 of the present studied patientsthere was a different substitution in the same codon inwhich tryptophan was replaced by serine (Trp582Ser).Mutations at codons Tyr568Asp, Ile571Thr, Thr574Tyr,Gln575Ile, Tyr578Phe, Asp579Gln, Asp579Pro,His580Leu, His580Tyr, His580Pro, Arg586Trp,Arg586Ile, Arg586Phe, Arg586Asp, Asn587Glu,Asn587Pro, Asn587His and Arg588Phe, Arg588Tyr, andArg588Lys have been reported [17,30,29,32]; however,in the present study we observed substitution mutations at Tyr568Ser, Thr574Pro, Gln575His, Tyr578Pro,Asp579His, His580Gln, Arg586Thr, Asn587Asp andArg588Met which have not been previously reported.We also detected 4 novel point mutations—Ile563Lys,Val569Leu, Tyr570Ser, and Pro577Ser—at codons 563,569, 570 and 577 respectively in exon 11 of the c-kit genewhich have not been previously reported in any neoplasiapatients. The mutations in exon 11 of the c-kit geneobserved in the present study between codons 550 and591 are in agreement with previously reported mutationsin different populations (Figure 2 and Table 2).

In conclusion, the present study is the first to reportthe presence of c-kit gene mutations in Indian leukemiapatients. The observed mutations in exon 11 of the c-kitgene may be involved in c-kit over expression in leukemia.These observations suggest that mutations in exon 11of the c-kit gene might be useful molecular genetic markersfor leukemia. Additional research with larger studygroups may clarify the prognostic implications of thesemutations, and their association with the pathogenesis andprogression of myeloid malignancy.

## CONFLICT OF INTEREST STATEMENT

The authors of this paper have no conflicts of interest, including specific financial interests, relationships, and/ or affiliations relevant to the subject matter or materials included.

## ACKNOWLEDGMENTS

This study was supported by an intramural grant fromEra’s Lucknow Medical College and Hospital, Lucknow,India.

## Figures and Tables

**Table 1:**
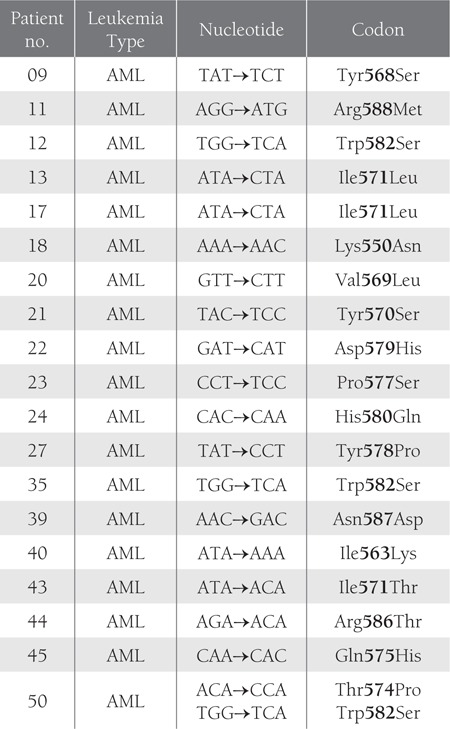
Point Mutations in Exon 11 of the c-kit gene

**Table 2:**
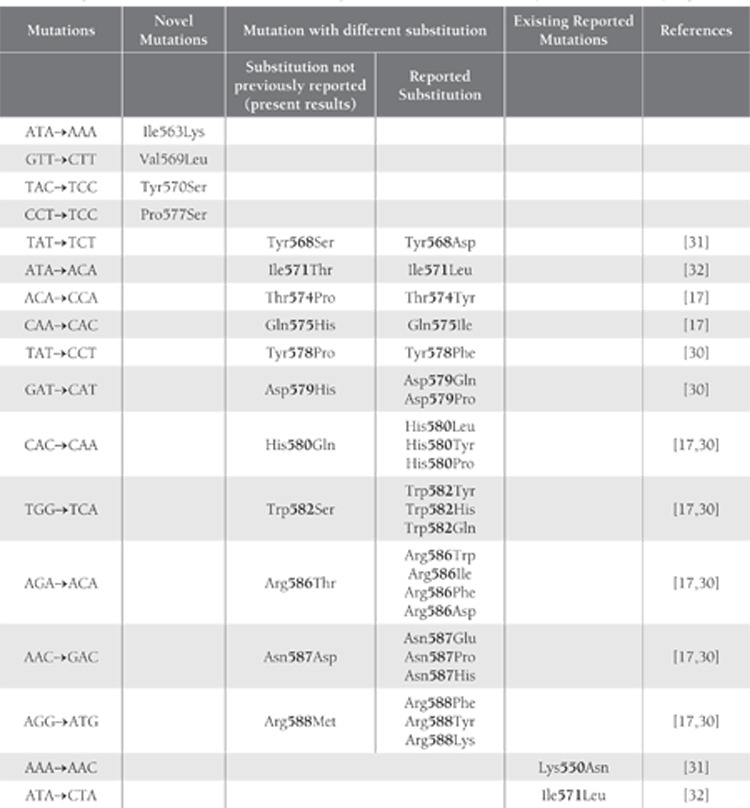
Comparison of Mutations in Exon 11 of the c-kit gene Identified in the Present Study and Those Previously Reported
